# Pathological analysis of cadavers for educational dissection by using postmortem imaging

**DOI:** 10.1111/pin.12857

**Published:** 2019-10-20

**Authors:** Sakon Noriki, Satoshi Iino, Kazuyuki Kinoshita, Yugo Fukazawa, Kunihiro Inai, Toyohiko Sakai, Hirohiko Kimura

**Affiliations:** ^1^ Division of Tumor Pathology, Department of Pathological Sciences, School of Medical Sciences University of Fukui Fukui Japan; ^2^ Division of Anatomy, Department of Morphological and Physiological Sciences, School of Medical Sciences University of Fukui Fukui Japan; ^3^ Division of Radiology, Department of Radiology and Laboratory Medicine, School of Medical Sciences University of Fukui Fukui Japan; ^4^ Division of Brain Structure and Function, Department of Morphological and Physiological Sciences, School of Medical Sciences University of Fukui Fukui Japan; ^5^ Division of Molecular Pathology, Department of Pathological Sciences, School of Medical Sciences University of Fukui Fukui Japan; ^6^ Autopsy Imaging Center, School of Medical Sciences University of Fukui Fukui Japan

**Keywords:** anatomy, educational dissection, pathological analysis, postmortem imaging

## Abstract

This study was performed primarily to clarify whether pathological analysis of cadavers for anatomical dissection is possible using postmortem imaging (PMI), and whether this is worthwhile. A total of 33 cadavers that underwent systematic anatomical dissection at our medical school also underwent PMI. Fixative solution was injected into the corpus 3–4 days after death. PMI was then performed using an 8‐slice multi‐detector CT scanner 3 months before dissection. Before dissection, a conference was held to discuss the findings of the PMI. First, two radiologists read the postmortem images without any medical information and deduced the immediate cause of death. Then, the anatomy instructor revealed the medical information available. Based on this information, the radiologist, anatomy instructor, and pathologists suggested candidate sampling sites for pathological examination. On the last day of the dissection period, the pathologists resected the sample tissues and processed them for pathological examination. In 12 of 33 cases, the presumed causes of death could be determined based on PMI alone, and revision of the cause of death described in the death certificate was considered in five (15.2%) cases, based on PMI and pathological analysis. This article presents a novel method of pathological analysis of cadavers for anatomical dissection using PMI without disturbing the anatomy education of medical students.

**Abbreviations:**
COD: cause of death
CT: computed tomography
HRP: horseradish peroxidase
MRI: magnetic resonance imaging
PMI: postmortem imaging
PBS: phosphate buffered saline

## INTRODUCTION

Anatomy, including dissection of human cadavers, is an indispensable basic subject in the medical education program.^1^, ^2^ In the recent medical education reform, there is a tendency for weight to be placed on clinical medicine and training and various alternative methods are being tried. Integration of newer teaching modalities and modern technology encourage interest and retention of anatomical knowledge and its clinical relevance.^3^ In anatomy in particular, it is intended that a medical student learns the normal physical structure, however, many cadavers used in anatomy harbor a variety of diseases making this difficult.

The cadaver can be analyzed using diagnostic imaging using computed tomography (CT) and/or magnetic resonance imaging (MRI). Postmortem imaging (PMI) seems to be the common term, however, the procedure is variously described as virtopsy in Switzerland,^4^ virtual autopsy in France,^5^ radio‐autopsy in Germany^6^ and autopsy imaging (Ai) in Japan.^7^

The imaging results of the cadaver are naturally included in the anatomy education for medical students. For example, in the Mount Sinai School of Medicine, the PMI of the cadaver is regarded as a powerful teaching tool and is standard in most anatomy courses,^8^ across various countries, including Ireland and Poland.^9^, ^10^, ^11^, ^12^, ^13^, ^14^ In Japan, many universities also perform PMI before anatomical dissection starts and combine imaging education with anatomy education.^15^ However, it is rare that any further medical examinations of the cadaver, other than imaging, are carried out.

While dissection of the cadaver is indispensable for the anatomical education of the normal physiological structure, it cannot be overemphasized that pathological autopsy is indispensable for the understanding of disease. The rate of academic autopsies has, however, been decreasing worldwide in recent years. Despite many benefits,^16^ autopsies are performed after less than 10% of all US deaths,^17^ and there has been a decrease in the hospital autopsy rate from 21.6% in 1990 to 7.9% in 1999.^18^ The mean hospital autopsy rate in 2013 in the UK was 0.69% of hospital deaths,^19^ and the autopsy rate in Germany is now below 10%.^20^ In France, the rate of autopsy in hospitals is also decreasing,^21^ while in Brazil, the median autopsy rate has fallen from 19.3% in 2003 to 10.6% in 2008 (*P* = 0.07).^22^ According to the Japan Council for Quality Health Care, the autopsy rate of 397 hospitals, which were tested by the hospital usability test in 2012, was 4.0%.^23^

Pathological examination of the cadaver for anatomical dissection is done incidentally, such as making specimens of the tumors found during dissection, since the primary purpose of the anatomical dissection is anatomy education. Thus, sampling a pathological specimen systematically has been impossible, because that would interfere with the anatomy education. Therefore, we attempted pathological analysis of cadavers with PMI for systematic anatomical dissection. One purpose of this study was to clarify whether pathological analysis of cadavers for anatomical dissection using PMI is possible. Furthermore, this paper shows the rate at which a more accurate cause of death could be determined.

## MATERIALS AND METHODS

### Objective

From October, 2010, to September, 2015, 100 cadavers underwent systematic anatomical dissection at Fukui University, School of Medicine. During their lifetime, permission for PMI was obtained from 33 of the donors.

Systematic anatomical dissection is conducted according to the Japanese law ‘A Body Donation Law’. The consent for PMI of the donated corpus was obtained from the bereaved individual. This study obtained approval for the research program from the Ethical Review Board of the University of Fukui, School of Medicine.

### Treatment of the corpus

Approximately 10 L of fixative solution (mixture of formalin, alcohol and glycerin) was injected into the corpus over 10–30 min through a femoral artery 3–4 days after death. Only the fixative solution was injected, and around 1 L was discharged (egested). The fixative seems to collect in the abdominal cavity and the thoracic cavity, thus, whether pathologically significant ascites and pleural effusion were present could not be determined. The brain was resected from the corpus 1 week after death because the gross neuroanatomy training of the resected brain is performed by a different part of the curriculum from the gross anatomy training of the human body. It is not possible to evaluate the brain histopathologically, since the brain is used for the gross neuroanatomy training of medical students.

### Taking PMI

The PMI was performed at the Ai center at the University of Fukui, 3 months before dissection, using an 8‐slice multi‐detector CT scanner (Hitachi Medico, Tokyo, Japan) used exclusively for autopsies, as described previously.^24^, ^25^, ^26^ The corpus was placed in the supine position, and a full‐body scan from the vertex to the toes was performed. The scanning conditions were 120 kV, 250 mA, 8 × 2.5 collimation, 1.125 pitch, 0.8 s rotation time, 5 mm slice thickness and 5 mm increments. No contrast agent was used in these cases. The corpus was appropriately treated with dignity at all times.

### PMI conference

Before anatomical dissection began, a PMI conference including two radiologists, an anatomy instructor, two pathologists and a radiological technologist was held. First, two radiologists (KK and TS) read the postmortem images of the corpus without any medical information and deduced the immediate cause of death. The algorithm for diagnosis is described in a previous article.^24^, ^25^, ^26^ Then, the anatomy instructor (SI) revealed the medical information. The medical information concerning the corpus included only the age, sex, cause of death and the place of death described in the death certificate. Based on this information, the radiologist, anatomy instructor and pathologists suggested candidate sampling sites.

### Conventional histopathology

Based on the PMI conference, the pathologists (SN and KI) resected the sample tissues from the lesions of the corpus on the last day of the dissection period. The sampled tissue from the lesion was sliced into 5 mm thick sections. The average number of samples was around four, one at the minimum and seven at the maximum, per case (Table 1). The tissues were embedded in paraffin and sectioned with a thickness of 6 μm. For routine histological examination, each section was stained with hematoxylin and eosin (H‐E). In a case with suspected amyloidosis or tuberculosis, Congo‐red staining or Ziehl‐Neelsen staining was performed, respectively.

**Table 1:** The summary of all cases that underwent pathological analysis by postmortem imaging

No.	Age (y)/ sex	PMI findings (Immediate cause of death by PMI)	Cause of death (COD) described in the death certificate:I. Immediate	Sampling site (number of samples)	Pathological findings and diagnosis	COD by total findings:I. Immediate COD
			CODII. Intermediate COD			II. Intermediate COD
			III. Underlying COD			III. Underlying COD
**1**	58/F	Air in vascular system and cerebrospinal cavity. CV port. Massive ascites. S/O cancerous peritonitis. Cyst or tumor in the left breast. Coarse calcification in the right breast. Calcified nodule in the thyroid gland. S/O Thyroid papillary carcinoma.	III. Breast cancer, bilateral.	Thyroid (3), ovaries (2), breast (1), intestine (1)	The colon wall has atypical epithelial cells from the submucosal layer to the serosal layer. This finding shows metastasis and cancerous peritonitis. These findings are compatible with metastatic breast cancer, but there is a possibility of metastatic gastric cancer.	I. Not determined
						III. Breast cancer and gastric cancer?
					In the thyroid gland, papillary carcinoma is found.	
					The cyst of the left breast has no epithelial cells. Cancer cells were not confirmed.	
					The left ovary is occupied by cancer cells similar to ones in the colon wall. Some are signet ring cells. These findings show Krukenberg tumor.	
		Swelling of bilateral ovaries, S/O metastases. Massive contents in the colon, S/O passage disorder. **(Not determined)**				
**2**	89/M	Air in the vascular system: portal vein, aorta, renal artery. Air also found in the peritoneal cavity, intestine, and stomach. Emphysema in the lung. Pleural effusion.	I. Drowning	Thyroid (1), lung (1)	Emphysema in the lung.	Not determined
					Adenomatous goiter in the thyroid gland.	I. Drowning
		**(Not determined)**				
**3**	73/M	Multiple nodules in the liver. S/O Metastases.	I. Cancerous peritonitis	Lung (4).	Papillary adenocarcinoma of the stomach. Invasion into lymph ducts, vessels, and peripheral nerves. Neutrophil infiltration surrounding tumor is noted.	I. Respiratory failure
			III. Gastric cancer			
						II. Pneumonia
		Little air in the vascular system. Ascites suspicious of peritonitis.			Emphysema in the lung. Hyaline membrane on alveolar spaces. Inflammatory cells with bacterial colonies in the alveolar spaces.	III. Gastric cancer.
		The wall of the antrum of the stomach is thickened. This finding suspicious of advanced cancer.				
		Infiltrative shadow in the lung. This finding suspicious of pneumonia.				
		Calcification of coronary arteries.				
		**(Not determined)**				
**4**	65/M	Pleural effusion, especially right‐sided. Air bronchogram in the right lung. Multiple nodules in the left lung. This finding suspicious of metastasis. Air in the peritoneal cavity.	I. Respiratory failure	Lung (4), Duodenum (3).	Multiple tumors in the lungs are composed of medium‐sized, atypical epithelial cells with necrosis. Immunostaining shows that tumor cells are positive for CK7, CEA, Chromogranin A, Synaptophysin, GCDFP‐15, CK5/6 (partially), and negative for CK20, CD56, androgen receptor, alpha‐amylase, S‐100, TTF‐1, p63. These findings are compatible with metastasis of parotid gland cancer. In the background of lung abscess and bronchopneumonia with bacterial colonies.	I. Not determined
			II. Lung metastasis, bilateral.			III. Parotid gland cancer
			III. Parotid gland cancer.			
		**(Respiratory failure)**				
**5**	91/M	Pleural effusion, cancerous pleuritis, cancerous peritonitis, Aortic aneurysm.	I. Cancerous pleuritis	Lung (2), pancreas (1), colon (1), mesentery (1).	Pancreas is severely degenerated and fibrous. Atrophic exocrine glands are seen in the fibrous pancreas.	I. Cancerous peritonitis and pleuritis.
			III. Pancreatic cancer			
						III. Pancreatic cancer.
					No distinct malignant findings are found in the removed tissue.	
		Cancer might be derived from the SMA area. This finding suspicious of pancreatic cancer				
					Fibrosis might be present	
					Stomach and major omentum are composed of well‐differentiated tubular adenocarcinoma. Cancer exposed on the serosa. In the colon, cancer nests are found on the serosa. Cancerous peritonitis.	
		**(Cancerous peritonitis and pleuritis)**				
					Metastatic tubular adenocarcinoma is noted at the subserosal area of the lung. Cancerous pleuritis.	
**6**	76/M	No gas (air) in the intestine.	I. Acute pneumonia	Lung (4), pancreas (1), spleen (1), bone marrow (1).	Pancreas is severely degenerated.	I. Septic shock S/O III. Leukemia
		Swollen pancreas suspicious of pancreatitis.				
		Infiltrative shadow of the lung suspicious of pneumonia.				
			II. Acute myelogenous leukemia			
		**(Not determined)**				
			III. Myelodysplastic Syndrome			
					Both lungs have hyaline membranes in alveolar spaces. This finding shows DAD.	
					Few leukemic cells in the removed tissue.	
					Hemophagocytosis, suspected cytokine storm and septic shock.	
**7**	85/M	Aneurysm in the arch of the aorta with calcification.	I. Pneumonia.	Lung (4).	Severe bronchopneumonia.	I. Pneumonia C/W
		Subtotal gastrectomy.				
		Severe emphysema.				
		**(Not determined)**				
**8**	94/F	Multiple liver nodules, liver metastases.	**I. Colon cancer.**	**Liver (1), colon (2), kidney (1).**	Multiple liver metastases, tubular adenocarcinoma.	**I. Hepatic failure**
						**II. Liver metastasis**
						III. Colon cancer
		The primary site is not determined.				
		Peritoneal dissemination.				
		Gall stones and nephrolithiasis.				
		Artificial joint in bilateral knee joints.				
		Femoral head fracture.				
		**(Hepatic failure due to metastasis)**				
					The entire circumference of the colon wall is swollen. Tubular adenocarcinoma. Cancerous peritonitis.	
					Femoral head fracture cannot be confirmed.	
**9**	69/M	Stent at the pyloric region of stomach → Advanced gastric cancer.	I. Cancerous peritonitis	Stomach (3), Sacral bone (1).	In the pyloric region of stomach, stent found as per imaging. Over all walls of stomach layers, signet ring cell adenocarcinoma is detected.	I. Cancerous peritonitis.
			III. Gastric cancer			
		Cancerous peritonitis.				
		In the right ilium, osteoblastic tumor.				
		Vertebral metastasis.				
		In oral cavity, implant and torus palatinus.				
					Cancer cells present from the muscular layer to the serosa. Cancerous peritonitis.	
		**(Cancerous peritonitis)**				
						III. Gastric cancer
					Bone marrow occupied by small tumor cells.	
					Epithelial binding is found and regarded as metastasis of the cancer, but seems to be different from the signet ring cells found in stomach.	
**10**	79/M	Postoperative state of chest wall.	I. Cerebellar infarction	Heart (4)	Myocardium shows no distinct infarction.	Not determined
		Stent in the coronary artery.				
		Bypass operation of the right coronary artery, anastomosis to a circumflex artery.				
					(No neutrophil infiltration in myocardium.)	
		The valve is also post‐replacement.				
		Gall stones. Artificial hip prosthesis.				
		**(Not determined)**				
**11**	72/M	Postoperative state of maxillary sinus.	I. Cancerous peritonitis.	Stomach (1), intestine (1).	No cancer cells found in the serosa of the colon. No cancerous peritonitis seen in the specimen of the colon.	Not determined
			III. Duodenal cancer.			
					However, the adipose tissue shows myxoid change, suspicious of cancer cachexia.	
		Severe subcutaneous edema.				
		Massive pleural effusion.				
		Postoperative state of stomach.				
		Emphysema, severe.				
		Metastatic lesion in the first lumbar vertebra				
		S/O metastasis of gastric cancer.				
		**(Not determined)**				
**12**	60/M	Large number of warts on the skin.	I. Brain stem glioma	Skin tumor (1)	Black thyroid, very hard.	Not determined
			II. Neurofibromatosis			
					Warts of the skin are neurofibromas.	
					(Stromal lymph ducts or capillaries were dilated.)	
		Calcification of the right lobe of the thyroid gland.				
		Left pleural effusion, encapsulated.				
		Old fracture of the rib.				
		Emphysema, severe.				
		Left inguinal testicular hernia				
		**(Not determined)**				
**13**	76/M	Emphysema.	I. Multiple myeloma	Soft tissue tumor (1), Bone marrow (1)	Osteolytic lesions composed of atypical plasma cells: Multiple myeloma (Plasmacytoma).	I. Not determined
		Osteolytic lesions in the bilateral ilium, right ischium、Left rib → metastasis.				
						III. Multiple myeloma
		Soft tissue tumor in the right femoral region → primary? Secondary? Lung or kidney suspected as primary site.				
					Soft tissue tumor of the right femoral region is amyloidoma.	
		**(Not determined)**				
**14**	86/F	Cardiac effusion → bloody → Cardiac tamponade	I. Acute cardiorespiratory failure.	Aorta (1)	Cancerous pericarditis. Adenocarcinoma.	**I. Cardiac tamponade**
					Immunostaining suspicious of metastatic lung cancer, but TTF‐1 negative.	
						**II. Cancerous pericarditis.**
			II. Lung metastasis.			
						III. Not determined.
			III. Ureteric cancer.			
		Pleural effusion also bloody.				
		However, seems to be no aortic dissection.				
		**(Cardiac tamponade)**				
**15**	78/F	Left parotid gland tumor → CT is uniform, and rise in concentrations → cyst‐related lesion S/O → Warthin's tumor.	I. Chronic heart failure.	Left parotid gland (1), lungs (2).	Warthin's tumor.	I. Bronchopneumonia?
					Bronchopneumonia. Organizing pneumonia.	
			II. Atrial fibrillation, pulmonary emphysema, severe anemia.			
		Distal part of the left clavicle and left femoral head fracture → trauma? No rib fracture.				
		Right pleural effusion with niveau → S/O bloody effusion. A small left pleural effusion.				
		Infiltrative shadow in the lung.				
		**(Not determined)**				
**16**	86/F	Calcification in the right eye ball.	I. Bleeding in the digestive tract.	Liver (2), Right lung (1), Right kidney (1).	Liver cirrhosis.	I. Not determined.
						III. Liver cirrhosis.
					Kidney, nothing particular.	
			II. DIC, Sepsis.		Micro‐thrombus in the lung, S/O DIC.	
			III. Pneumonia.			
		Right nephrolithiasis.				
		Cysts of the left kidney.				
		Osteoarthroplasty of the hip joint.				
		Liver cirrhosis, S/O.				
		**(Not determined)**				
**17**	84/F	No thyroid glands, S/O Post total thyroidectomy	I. Pneumonia.	Left cardiac ventricle (2), Right cardiac ventricle (1), Lung (2).	Severe dilatation of left cardiac ventricle.	I. Sepsis?
					Micro abscesses in the myocardium.	
		Cardiac pacemaker.				
		Cardiomegaly.				
		Severe calcificat				
		ion of the pulmonary upper lobes.			Calcification of the lung.	
		Nothing particular in the abdominal cavity.				
		Artificial joint (prosthesis) in the left hip joint.				
			II. Old tuberculosis and chronic obstructive respiratory disease.			
		**(Not determined)**				
**18**	105/F	Gall stones. Dilatation of intrahepatic bile ducts and gallbladder.	I. Chronic heart failure.	Pancreas (2), mesentery (1)	Intraductal papillary mucinous neoplasm (IPMN) of the pancreas.	Not determined
			II. Died of old age.			
		Tumor of the pancreatic head, S/O pancreatic cancer. S/O obstructive jaundice.				
					No atypical cells in the serosa of the stomach. No finding of cancerous peritonitis.	
		Tumor of the pancreatic tail.				
		Cancerous peritonitis suspected.				
		Abdominal aortic aneurysm (AAA) with mural thrombosis.				
		Interstitial pneumonia, carcinomatous lymphangitis of the lung.				
		**(Not determined)**				
**19**	85/F	The lung is diffusely infiltrative. Especially right lung is focally distinct. S/O aspiration pneumonia.	I. Acute myelogenous leukemia	Lung (3),	Small cell carcinoma of the lung, S/O. Tumor cells were positive for CD56, negative for chromogranin A and synaptophysin. TTF‐1 and Ki‐67 (MIB‐1) also negative.	**I. Respiratory failure**
						II. Pneumonia
		Right pleural effusion.				
		Dilatation of ascending colon, S/O ileus.				
		Osteolytic lesion in the sacrum.				
		Artificial prostheses of bilateral knee joints.				
		Right adrenal gland swelling				
		Accessory spleen.				
		**(S/O aspiration pneumonia)**				
					Pulmonary edema and pneumonia.	
					Atypical cells in the spleen, C/W leukemic cells.	
					Hemosiderosis of the spleen.	
					Sacral lesion not detectable.	
				spleen (1).		
						III. Leukemia
**20**	88/F	Full dentures, but not metal. Pleural effusion, Right > Left. Mediastinal lymph nodes markedly swollen.	I. Malignant lymphoma	Lung (3).	We were not able to take mediastinal lymph nodes, but took hilar lymph nodes of the right lung. Tumor cells were positive for CD15 and CD30. → Hodgkin’s lymphoma.	**I. Respiratory failure**
						III. Hodgkin’s lymphoma
					Bronchopneumonia.	
		Little gas (air) in the digestive tracts. Simple cyst with hemorrhage in the left kidney.				
		Artificial joint (prosthesis) of the right knee joint.				
		**(Not determined)**				
**21**	86/F	Infiltrative shadow of the lung. No pleural effusion.	I. Aspiration pneumonia	Lung (2),	Pneumonia. Pulmonary edema.	I. Respiratory failure
					Dilatation of blood vessels in the gastric wall. Stones in the kidney. The kidney tissue surrounding stones is infectious.	
				kidney (2), stomach (1).		
					A large stone in the urinary bladder, 5.5 cm in diameter.	
		Marked air in the abdomen. Mesentery and gastric wall also had air. S/O postmortem changes.				
		Large stones with lamellar structure in the kidney and urinary bladder. Gall bladder stones.				
		**(Pneumonia)**				
**22**	80/F	Infiltrative shadow of the lung. S/O pneumonia.	I. Pneumonia	Pancreas (2),	Aspiration pneumonia, Pulmonary edema.	I. Respiratory failure
						II. Aspiration pneumonia
					Cannot clarify pancreatitis.	
					Macroscopic fracture of the distal part of the left clavicle.	
				lung (2).		
		Swelling of the pancreas, and ascites → S/O acute pancreatitis.				
		Fracture of the distal part of the left clavicle.				
		**(Pneumonia or acute pancreatitis)**				
**23**	69/F	The lung was relatively clear. Infiltrative shadow in the back side of the lung. Severe emphysema with fluid. S/O pneumonia or water.	I. Gall bladder cancer	Lung (2),	Severe emphysema.	I. Respiratory failure
						II. Emphysema and cancer metastasis
					Metastatic tumor in the lung, 3.5 cm in diameter. Tumor is composed of moderately differentiated adenocarcinoma, C/W metastatic gallbladder cancer.	
						III. Gall bladder cancer
				Kidney (2),		
					Cannot confirm multiple nodules in the liver. Only congestion of the liver.	
				Stomach (1).		
					Bacterial colonies considered to have grown in the liver after death. This might result in air in the liver.	
		Air in the digestive tract. S/O pneumatosis cystoides intestinalis.				
					Metal device in the stomach. (According to what medical student said)	
		Multiple nodules in the liver, liver metastases. Air in the portal vein of the liver was like angiography.				
		Surgical staples (metal) in the stomach. S/O post‐operative state of stomach.				
		**(S/O Pneumonia)**				
**24**	76/M	Left infiltrative shadow, S/O pneumonia.	I. Glioblastoma	Lung (3),	Pneumonia, Pulmonary edema.	Not determined
					Thrombus in the blood vessels of the lung. S/O DIC.	
					Cavity in the right upper lobe. C/W Aspergillosis.	
					Fatty liver. Congestion of the liver, bile stasis in the liver. S/O jaundice.	
				Liver (1),		
					Cannot confirm the pneumatosis. No sign of peritonitis.	
				Transverse colon (1).		
		Left pleural effusion.				
		In the upper lobe of the right lung, cavity is noted. S/O Aspergillosis. Bulla in the lower lobe.				
		Free air in the abdominal cavity. Ascites. → S/O peritonitis.				
		Fatty liver.				
		Pneumatosis cystoides intestinalis in the transverse colon.				
		**(S/O Pneumonia)**				
**25**	69/F	Infiltrative shadow of the lung, Right > Left.	I. Died of old age	Lung (3).	Three tissues were sampled from the right lung. In all three tissues, caseating granulomas were found. S/O tuberculosis.	Not determined
					And neutrophil infiltration was also seen (and Pneumonia)	
		The shadow of the left lung was like a surface of a muskmelon → S/O congestion.				
		Cavity, pneumonia, and abscess in the upper lobe of the right lung → Bulla and Infection?				
		Calcification of pleura.				
		Pleural effusion.				
		Small gallbladder stones.				
		Cyst of the right kidney.				
		Stones of the left kidney.				
		**(S/O pneumonia)**				
**26**	95/F	Bilateral pleural effusion.	I. Chronic heart failure	Lung (2), Mediastinal lymph nodes (2).	Pulmonary edema, Pneumonia.	Not determined
		Esophageal hiatal hernia				
		Calcification of the mitral valves.				
		Free air in the abdominal cavity.				
		**(Not determined)**				
**27**	83/F	S/O bilateral pleural effusions.	I. Pancreatic cancer	Lung (2), pancreas (2),	Pneumonia, S/O aspiration pneumonia.	I. Respiratory failure
						II. Aspiration pneumonia
					Dilatation of the blood vessels in the gastric wall. Gastric emphysema seemed to be postmortem change.	
				stomach (1).	Poorly differentiated adenocarcinoma in the pancreas. Peripheral nerve invasion is found.	
		Infiltrative shadow → Pneumonia, S/O aspiration pneumonia.				
		Emphysema in the wall of the stomach, gastric emphysema.				
		Large gallbladder.				
		Fracture of the femoral head.				
		**(Not determined)**				
**28**	87/M	Marked calcification of the aorta.	I. Acute pneumonia	Lung (1).	Severe calcification of the aorta.	Not determined
					Calcified nest in the right lung, 2.0 cm in diameter.	
			II. Dysphagia		Pulmonary edema.	
					Metal clip in the stomach. (According to what medical student said)	
			III. Cerebral infarction			
		Multiple calcified lesions in the right lung. S/O calcified granuloma. S/O pneumonia of the right lung. Emphysema, mild in degree. A small pleural effusion. Metal clip or foreign body in the stomach.				
		**(Not determined)**				
**29**	89/M	Nasal polyp or inverted papilloma in the nasal sinus.	I. Acute heart failure	Thyroid (2),	Adenomatous goiter of the left lobe of the thyroid.	Not determined
					Oxalate crystals in the thyroid follicle.	
					Small granuloma without necrosis in the left lung. S/O sarcoidosis, or hypersensitivity pneumonitis.	
				lung (2).		
					Bone marrow embolism in the lung. S/O by the resuscitation technique.	
		Calcification in the left lobe of the thyroid gland.			Calcification of the aorta.	
		Infiltrative shadow of bilateral lungs. → Pneumonia.				
		Dilated stomach and digestive tracts. S/O Ileus.				
		Prostatic hyperplasia.				
		**(Not determined)**				
**30**	100/F	Pleural effusion and infiltrative shadow of the back side of the bilateral lungs and postmortem changes.	I. Died of old age	Lung (3),	Infectious renal cyst, pyonephrosis, perinephric abscess.	**I. Sepsis S/O**
						III. Perinephric abscess
					→ S/O Sepsis.	
					Pleural calcification.	
				kidney (3).		
			II. Congestive heart failure			
			III. Hypertension			
		Calcification of the right pleura.				
		Cysts of the left kidney. The content is partially whitish.				
		**(Not determined)**				
**31**	97/F	Pleural effusions in both pleural cavities. Infiltrative shadow in the back sides of both lungs.	I. Multiple cerebral infarctions	Lung (2).	Squamous cell carcinoma of the right lung, S3.	Not determined
					Fibrinoid pleuritis, right‐sided.	
		Nodule in S3 of the right upper lobe. Tumor or inflammation.				
		Cyst of the left kidney.				
		Post fracture of the left femoral head, false joint formation.				
		**(Not determined)**				
**32**	78/M	Pleural effusions in both pleural cavities.	I. Lung cancer	Lung (2).	Adenocarcinoma of the left lung hilar region.	I. Respiratory failure
						III. Lung cancer
		Infiltrative shadow in the back side of the both lungs. Left » Right				
		Nodule in the left pulmonary hila → Lung cancer and Pneumonia.				
		Emphysema.				
		Free air in the abdominal cavity. The cause of pneumoperitoneum is unknown. S/O artificial at fixation.				
		**(Respiratory failure due to left lung cancer and pneumonia)**				
**33**	89/M	Pacemaker	I. Aspiration pneumonia	Lung (2).	Pulmonary edema.	Not determined
		Pleural effusions in both pleural cavities.				
		Infiltrative shadow in the back side of both lungs.				
		Marked emphysema and pneumonia.				
		No abdominal findings.				
		Diverticulum in the right side of the urinary bladder, 5 cm in diameter.				
		**(Respiratory failure)**				

Abbreviations: COD, cause of death; C/W, compatible with; DAD, diffuse alveolar damage; PMI, postmortem imaging; S/O, suspicious of.

### Immunohistochemical staining

Immunostaining and special staining were performed as needed. The method of immunostaining in our laboratory has been described previously.^27^, ^28^ That is, 4 μm thick sections of paraffin‐embedded tissue were deparaffinized with xylene, which was then replaced with ethanol. After washing with water, the intrinsic peroxidase activity was blocked with a 0.03% H_2_O_2_ solution dissolved in absolute methanol at room temperature for 15 min, and the section was then rinsed with phosphate buffered saline (PBS). The sections in PBS were heated in a microwave oven at 750 W for 15 min for heat‐mediated antigen retrieval. The sections were then reacted at 4°C overnight with the antibody, which was diluted according to the directions for use. All antibodies were commercially purchased: CK5/6, TTF‐1, p63, AE1/AE3 (Nichirei Biosciences, Tokyo, Japan), CK7, CK20, CD15, CD30, CEA, S‐100, PSA, AFP, EMA, p40 (Agilent, Glostrup, Denmark), Chromogranin A, Synaptophysin CD56, GCDFP‐15 (Leica Biosystems, Wetzlar, Germany). Detection of antibody reactivity was performed using a commercially available kit (Dako EnVision Plus‐horseradish peroxidase (HRP); Agilent, Glostrup, Denmark), as follows. The sections were reacted with a goat‐anti‐mouse EnVision‐HRP–enzyme conjugate for 30 min at room temperature, followed by rinsing with PBS. The peroxidase color reaction was visualized by incubating the sections with the chromogen, 0.02% 3‐3‐diaminobenzidine tetrahydrochloride (Sigma Chemical Co., St Louis, MO, USA), at room temperature for 10 min. The sections were then washed with water. After the immunostaining procedures were completed, the sections were lightly counterstained with hematoxylin. As a negative control, the exact same procedure was performed without the primary antibody. The pathological diagnosis was done based on these procedures, and the specimens were analyzed in the pathology laboratory.

## RESULTS

The summary of all cases that underwent pathological analysis is shown in Table 1. In 12 (36.4%) of 33 cases, the presumed causes of death could be determined based on PMI alone (Case Nos. 4, 5, 8, 9, 14, 19, 22, 23, 24, 25, 32 and 33). In 5 (15.2%) of 33 cases (Case Nos. 8, 14, 19, 20 and 30), revision of the cause of death described in the death certificate was required based on PMI and pathological analysis. New findings that were not described in the death certificate included pneumonia, pulmonary emphysema, cirrhosis, liver metastasis of cancer, Warthin’s tumor and lung cancer.

Of these cases, three interesting cases are presented below.

Case 13 **A 76‐year‐old man**

**Figure 1:**
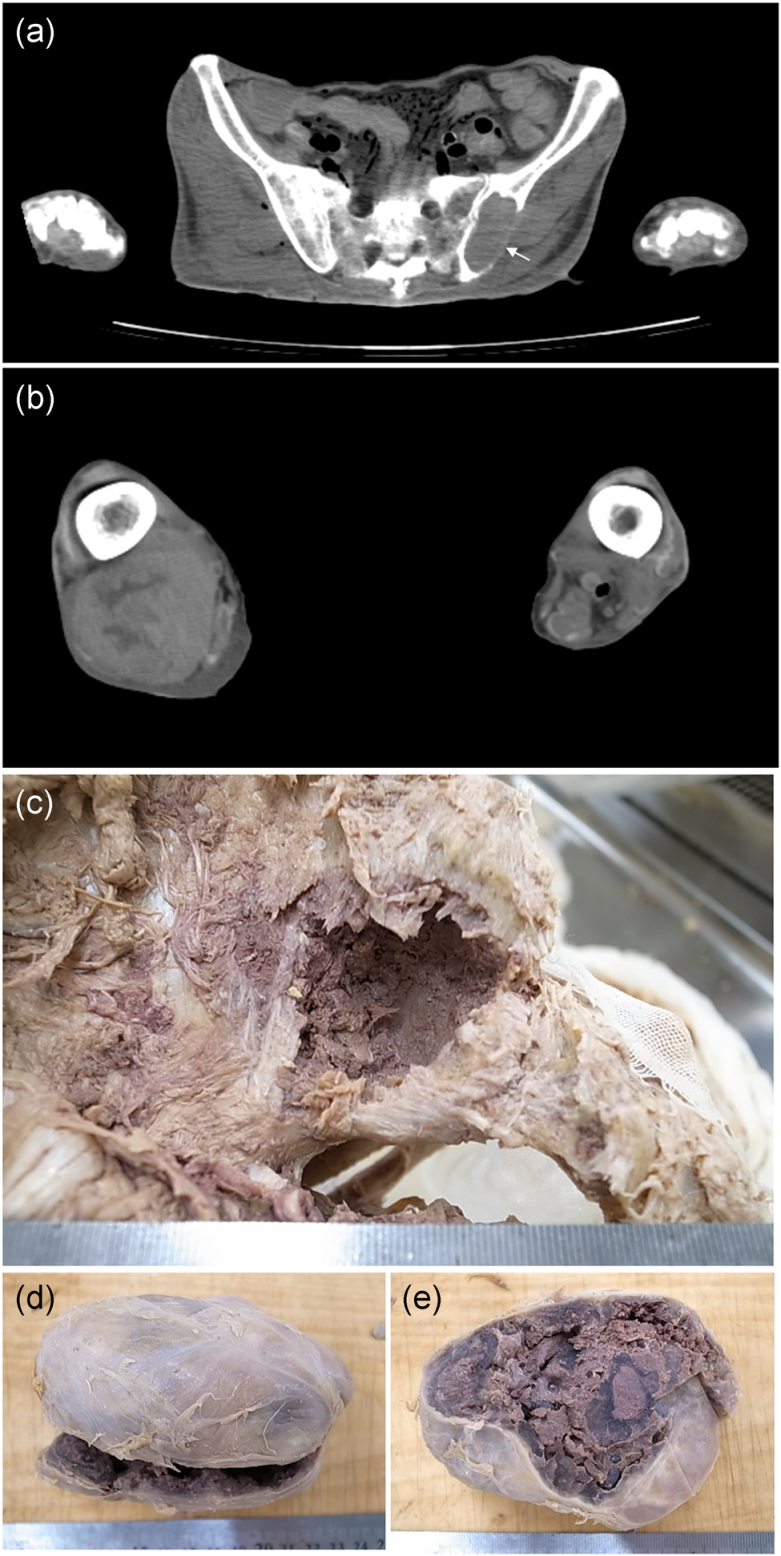
Postmortem imaging and gross appearance of anatomy Case No. 13. PMI‐CT shows nodular osteolytic change (arrow) of the left iliac bone (**a**) and a subcutaneous nodule of the right femoral posterior region (**b**). Macroscopic view of the osteolytic lesion of the left ilium (**c**) and removed tumor of the right femoral posterior region (**d**) at the time of anatomy dissection. PMI‐CT, postmortem imaging‐computed tomography.

**Figure 2:**
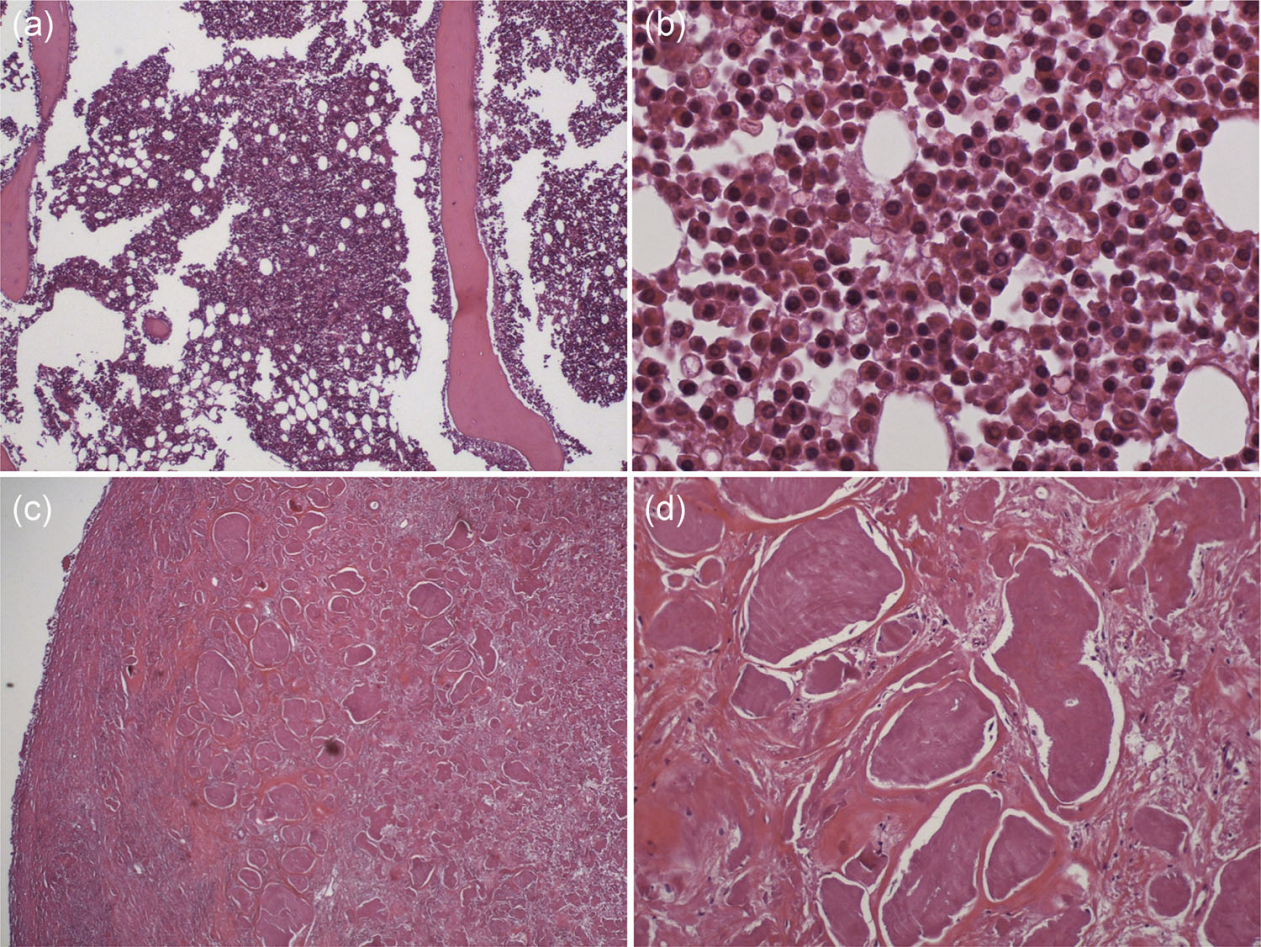
Histopathological findings of iliac bone and femoral posterior soft tissue tumor Case No. 13. The bone marrow shows a hypercellular bone marrow (**a**), and the bone marrow is occupied by monotonous plasma cells (**b**). Tumor of the femoral posterior region consists of amorphous eosinophilic materials (**c**). The nodule has no cellular elements, but extracellular, eosinophilic, proteinaceous deposits (**d**), which later were stained with Congo‐red and show apple‐green birefringence under polarization. All images show hematoxylin and eosin (HE) staining.

Osteolytic lesions in the bilateral ilium (Fig. 1a), right ischium and left rib were found on PMI. These findings suggested metastasis of a malignant tumor. In addition, a soft tissue tumor was also noted in the right femoral region (Fig. 1b). Whether this soft tissue tumor was primary or secondary was an issue for discussion at the PMI conference. The cause of death given on the death certificate was multiple myeloma. Iliac bone (Fig. 1c) and a soft tissue tumor (Fig. 1d) were obtained, and pathological specimens were prepared. These pathological specimens showed atypical plasma cells in the bone marrow of the ilium (Fig. 2a,b), and the soft tissue tumor consisted of eosinophilic amorphous material (Fig. 2c,d). The material showed apple‐green birefringence with Congo‐red staining. The material was thus amyloid deposits, and the soft tissue tumor was diagnosed as amyloidoma.

Case 14 **An 86‐year‐old woman**

**Figure 3:**
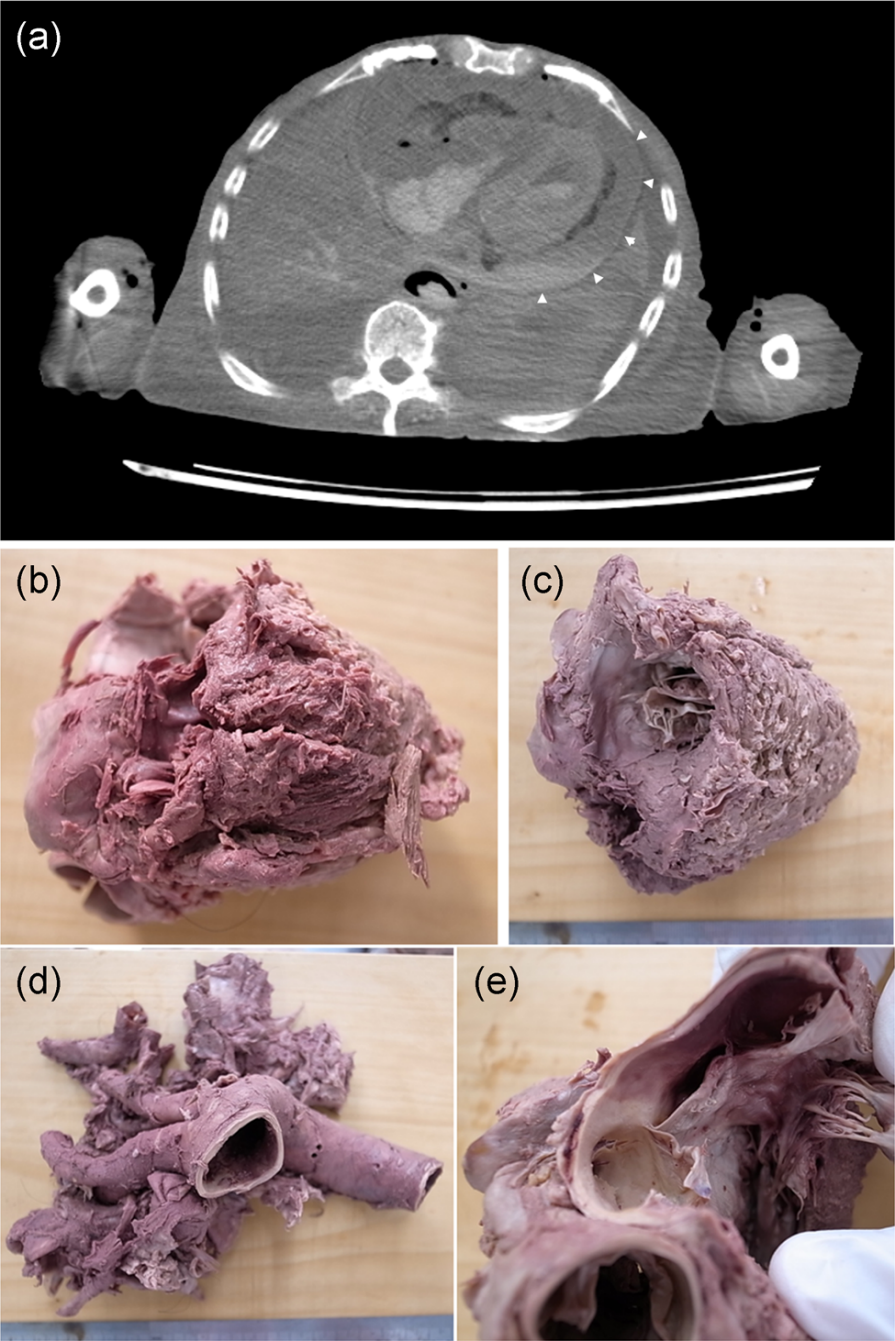
Postmortem imaging and gross appearance of anatomy Case No. 14. PMI‐CT shows a pericardial effusion (**a**, arrow heads). The pericardial fluid forms a niveau (horizontal surface) that suggests bloody fluid. The macroscopic view of the heart shows no cardiac wall rupture on the posterior side (**b**) or the anterior side (**c**). There is no dissection of the aortic root (**d**). The pathology specimen was obtained from the aortic root (**e**). PMI‐CT, postmortem imaging‐computed tomography.

**Figure 4:**
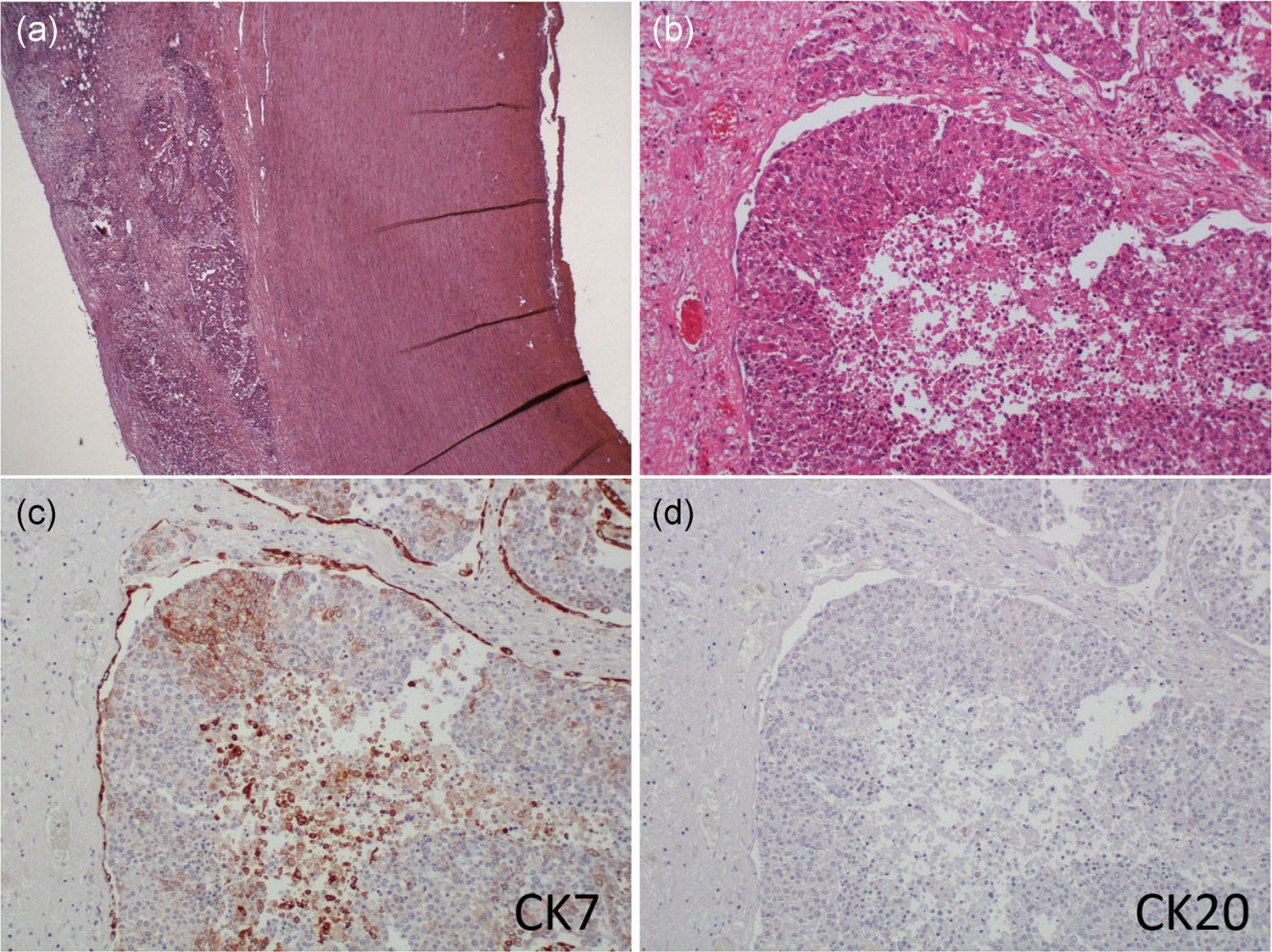
Histopathological findings of the aortic root.Metastatic cancer is found in the adventitia of the aortic root (**a**). The cancer shows solid growth, and some cancer nests have central necrosis (**b**). Both specimens were stained with hematoxylin and eosin. The immunostaining for CK7 is positive (**c**), but CK20 is negative (**d**). Both specimens were counterstained with hematoxylin.

On PMI, a cardiac effusion was found. The effusion was suspected to be bloody (Fig. 3a), so cardiac tamponade was the suspected cause of death. However, there seemed to be no rupture of a cardiac wall or aortic dissection (Fig. 3b–e). The aortic root was obtained for pathological examination. The pathological specimens showed cancer metastasis in the adventitia of the aortic root and pericardium (Fig. 4a,b). In order to determine the origin of the cancer, immunostaining was performed. Cytokeratin 7 was positive, but cytokeratin 20 was negative (Fig. 4c,d). Though lung adenocarcinoma was suspected, immunostaining for TTF‐1 was negative (data not shown). The cause of death on the death certificate was acute pulmonary vascular failure. However, the examination showed cardiac tamponade as the immediate cause of death, and cancer metastasis as the cause of the cardiac tamponade.

Case 20 **An 88‐year‐old woman with Hodgkin’s lymphoma**

**Figure 5:**
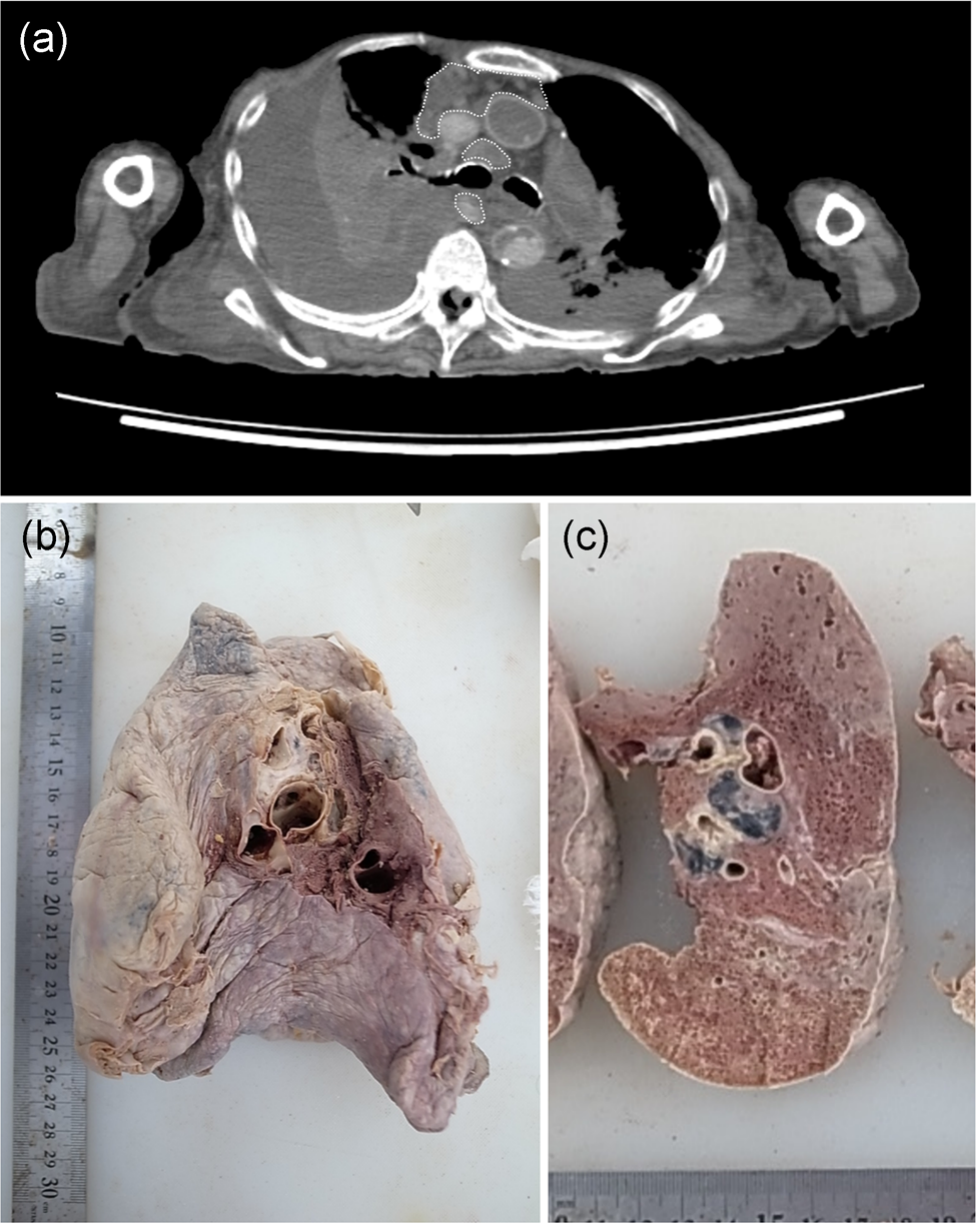
Postmortem imaging and gross appearance of anatomy Case No. 20. Chest PMI‐CT shows that mediastinal lymph nodes and right hilar lymph nodes are markedly swollen (dotted line area) (**a**). A pleural effusion with right‐sided predominance is also seen. The macroscopic view of the right lung (**b**) and the cut surface of the right lung at the hilar level (**c**). A swollen hilar lymph node is seen. PMI‐CT, postmortem imaging‐computed tomography.

**Figure 6:**
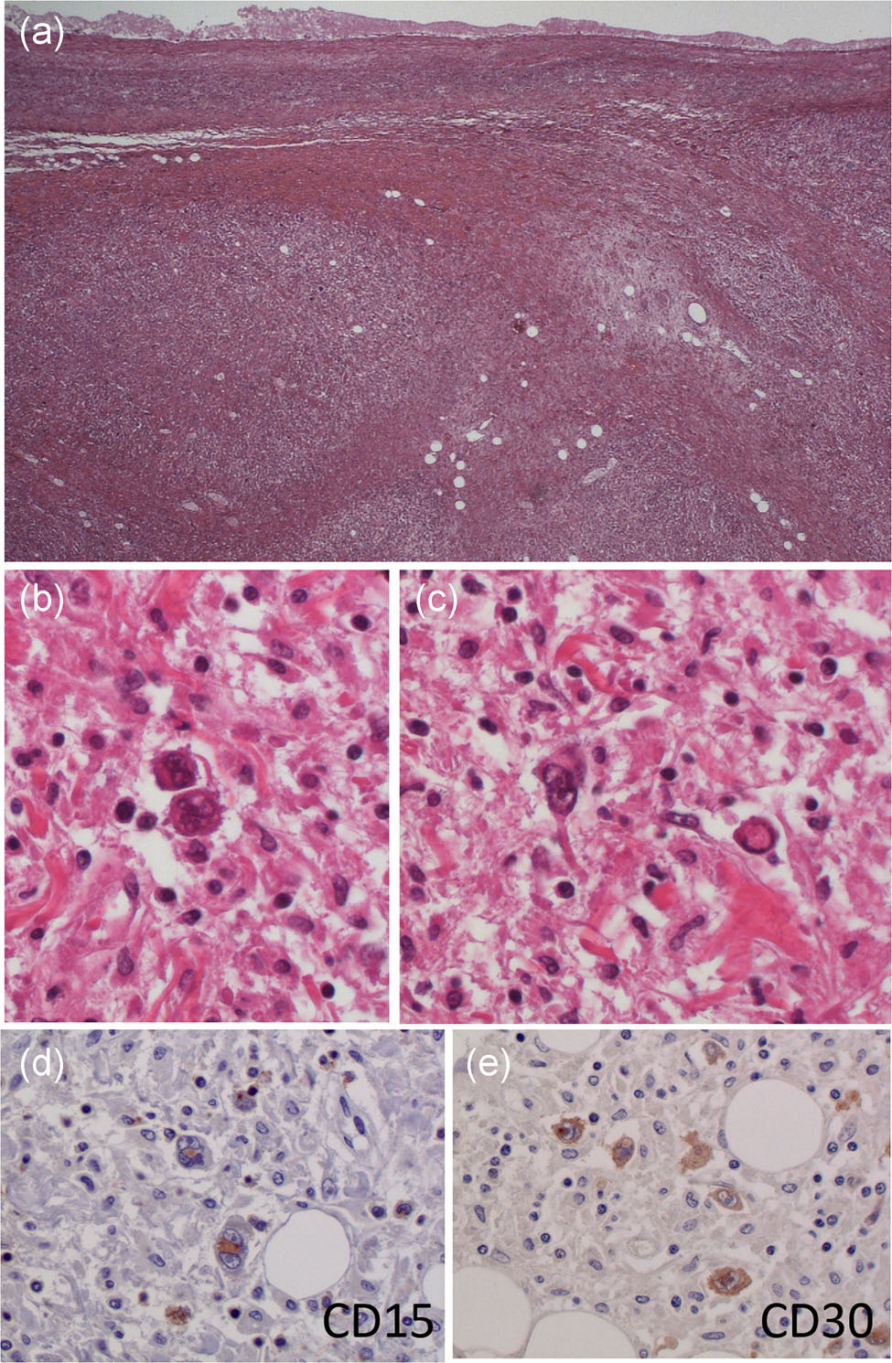
Histopathological findings of the right hilar lymph node. In the specimens of the pulmonary hilar lymph nodes, lymph follicular structure is not preserved, but nodular fibrosis is seen (Fig. 6a). Some multinucleated giant cells are found in the background of small lymphocytes and fibroblasts (Fig. 6b,c). Some nuclei have prominent nucleoli, and binucleated Reed‐Sternberg (R‐S) cells are also found. The immunostaining shows that the binucleated R‐S cells are positive for CD15 and CD30 (Fig. 6d,e). With these findings, the diagnosis is Hodgkin’s lymphoma.

Bilateral pleural effusions (right > left) were noted on PMI. Mediastinal lymph nodes were markedly swollen (Fig. 5a). However, mediastinal lymph nodes could not be obtained, but pulmonary hilar lymph nodes were taken instead (Fig. 5b,c). On examination of the specimens of the pulmonary hilar lymph nodes, the lymph follicular structure was preserved, but nodular fibrosis was seen (Fig. 6a). Lymphocytes were infiltrating into the surrounding adipose tissue. Some multinucleated giant cells were found in the background of small lymphocytes and fibroblasts (Fig. 6b,c). Some nuclei had prominent nucleoli, and binucleated Reed‐Sternberg cells were also found. Hodgkin’s lymphoma was suspected, and immunostaining was performed. The immunostaining showed that the binucleated Reed‐Sternberg cells were positive for CD15 and CD30 (Fig. 6d,e), and Hodgkin’s lymphoma was diagnosed.

## DISCUSSION

Although the cadavers for anatomical dissection were in the fixed state, interpretation of PMI was more valuable than expected. It was possible to diagnose cardiac tamponade (Case No. 14) and tumor or nodular lesions (Case Nos. 1, 3, 8, 11, 13, 15, 20, 23, 29, 31 and 32) with PMI, similar to clinical imaging. With respect to ascites and pleural effusions, since the possibility of effusion of the fixing solution could not be ruled out, the evaluation was difficult.

Furthermore, with respect to the specimens made from the cadavers, not only hematoxylin and eosin staining, but also some immunostaining was possible. On immunostaining, reactivity of CK‐7, CK‐20, CD30, CD15, CD56, CEA, Chromogranin A, Synaptophysin, GCDFP‐15, and CK5/6 was obtained, but TTF‐1, Ki‐67, androgen receptor, alpha‐amylase, S‐100, and p63 did not react. There might be a difference between cell surface antigens and nuclear antigens. A further examination of the reactivity of immunostaining of pathology specimens from cadavers for anatomical dissection may be required.

The total number of tissues and organs for histopathological evaluation might seem to be very small (from 1 to 7 only). The pathologists (SN and KI) sampled only the site which had abnormal findings on PMI in the PMI conference, and there was a time (temporal) limitation to have to obtain sample tissues and organs from many of the cadavers on the final day of the anatomy practice for the medical student. The pathologists knew the findings of the death certificate and PMI in PMI conference, and they therefore performed a minimum sampling based on these findings.

The death certificate of Japan is significant for two main reasons: (i) it proves human death medically and legally, and (ii) it provides mortality statistics by cause of death . However, the cause of death information obtained from death certificates is often inaccurate and incomplete. The accuracy of the underlying cause of death recorded on death certificates when multiple diseases are present in Japan has been discussed.^29^ The concordance rate of major underlying cause of death (cancer, heart disease and pneumonia) reported on death certificates compared with a reference standard of pathologist assessment based on autopsy data and clinical records was evaluated. The results of this article were ‘The concordance rate was relatively high for cancer (81%) but low for heart disease (55%) and pneumonia (9%).The overall concordance rate was 48%’. On the other hand, of the 33 cases in our study, the cause of death of 11 cases was not able to be determined by PMI findings and pathological analysis. Five cases (Case Nos.14, 15, 16, 17 and 30) had discrepancies among cause of death (COD) in the death certificate and COD by PMI findings and pathological analysis. The concordance rate was 77% (17 cases / 22 cases).

As for as Case 14, the discrepancy between COD in the death certificate and COD by total findings was explained above. Although heart failure should be avoided as the immediate COD on the death certificate, the immediate COD in the death certificate of Case 15 was chronic heart failure. We considered bronchopneumonia as the COD from the total findings. The underlying COD in the death certificate of Case 16 was pneumonia, but the liver pathologically showed liver cirrhosis. The immediate COD in the death certificate of Case 17 was pneumonia, but micro abscesses in myocardium were found. We suspect that sepsis was more accurate as the COD than pneumonia. As for as Case 30, we doubted that the 69 year‐old‐ woman had ‘died of old age’ as recorded in the death certificate, however, pathological analysis revealed infectious renal cyst, pyonephrosis and perinephric abscess. Sepsis was suspected as COD from these findings.

Of the 33 cases, it was possible to presume (or guess) the causes of death from PMI alone in 12 (36.4%). This is almost equivalent to the rate of determining the cause of death from PMI alone in forensic police‐related cases.

Throughout this series of pathological analyses, there were some interesting cases. Amyloidoma of the right femoral region was diagnosed in Case No. 13 by combined PMI and pathological examination. Soft tissue amyloidoma is a rare condition that presents primarily in the abdomen and/or mediastinum and more uncommonly on the extremities.^30^, ^31^, ^32^ There have been only a handful of case reports since 1998. We were able to see an interesting case by doing a pathological analysis using PMI of cadavers for anatomical dissection.

In Case 14, although pericardial tamponade as the cause of death was diagnosed by PMI, the cause of the pericardial tamponade was not found. On pathological analysis using immunostaining, it was possible to deduce the cause of death in greater detail. Even in Case 20, it was possible to diagnose the Hodgkin’s lymphoma correctly by doing the pathological analysis with immunostaining.

By performing PMI on cadavers for anatomical dissection and analyzing the pathological findings, it was possible to perform a pathological anatomy examination similar to what is called ‘minimally invasive autopsy’.^33^ In some cases, it was possible to identify interesting findings and understand the cause of death in more detail.

Moreover, it is thought to be important that students understand therapeutic signs and the pathological lesions of the cadaver when doing anatomical dissection to learn normal anatomy.

It is needless to say that pathological autopsy is very important to investigate the cause of death and the pathophysiological analysis of disease. However, a falling autopsy rate has been noted for quite a long time. The tendency for a decrease in the autopsy rate began in about the 1990s. In our hospital, there were 88 autopsies a year in 1989, which decreased to 34 a year in 2010. On the other hand, educational autopsies for medical students are carried out on about 25 corpuses every year. The number of autopsies of 25 a year corresponds to approximately 74% of the pathologic autopsies at our hospital in 2010.

As part of anatomy education, autopsy training is performed to study normal anatomy, but the cadavers dissected are never ‘normal’ humans. If a ‘normal’ human is necessary, only a simulator should be used. We also believe that it becomes one of the purposes of anatomy training to realize that the corpus being dissected was a human who suffered from illness when alive. In this way, the pathological perspective is required also in anatomy training.

A novel method of pathological analysis of cadavers for anatomical dissection using PMI without disturbing the anatomy education of medical students was presented in this paper. The pathological specimen obtained using PMI was able to be used not only for hematoxylin and eosin (HE) staining, but also some immunostaining. With this new method, it was possible to infer the cause of death in approximately 36.4% of cases. This method seems to be useful for understanding the pathophysiology and cause of death for not only the medical student, but also the pathologist.

## DISCLOSURE STATEMENT

None declared.

## AUTHOR CONTRIBUTIONS

Conception and design of the study: SN. Taking PMI of the corpus and instruction of anatomy dissection training: SI, YF. The interpretation of the PMI: KK, TS, HK. Sampling from the corpus and pathological diagnosis: SN, KI. Drafting the manuscript or figures: SN.
